# Level of Vascular Ligation for Sigmoid Colon Cancer: Does It Matter? The Pina Low‐T Study

**DOI:** 10.1002/jso.70291

**Published:** 2026-05-26

**Authors:** Silvia Picotto, Elisabetta Seno, Marcello Calabrò, Massimiliano Di Marzo, Giuseppina Lo Moro, Mauro Santarelli, Paolo Delrio, Andrea Muratore

**Affiliations:** ^1^ Department of Surgical Sciences University of Torino Turin Italy; ^2^ Surgical Department “E. Agnelli” Pinerolo Hospital Pinerolo Italy; ^3^ Colorectal Surgical Oncology, IRCCS “Fondazione G. Pascale” Naples Italy; ^4^ Department of Public Health Sciences and Paediatrics University of Turin Turin Italy; ^5^ Department of General and Emergency Surgery A.O.U. Città della Salute e della Scienza di Torino Torino Italy

**Keywords:** anastomotic leak, inferior mesenteric artery ligation, lymphadenectomy in colon surgery, oncological outcomes in sigmoidectomy, sigmoid colon cancer, vascular preservation

## Abstract

**Background and Methods:**

In sigmoid colon cancer surgery, the inferior mesenteric artery (IMA) can be ligated at its origin (high ligation, HLG) or distal to the left colic artery bifurcation (low ligation, LLG). While high ligation facilitates lymph node harvest and mobilization, it may compromise colonic perfusion and increase nerve injury risk. Low ligation preserves the LCA and may improve anastomotic blood supply. We conducted a retrospective multicenter study including patients who underwent sigmoidectomy for sigmoid colon cancer between January 2017 and December 2022. Short‐ and long‐term outcomes were compared between HLG and LLG.

**Results:**

A total of 185 patients were included (127 HLG, 58 LLG). Median postoperative length of stay was similar (6 vs. 5 days; *p* = 0.879). Anastomotic leak rates were 5.5% in HLG and 1.7% in LLG (*p* = 0.438). Patients undergoing LLG had a higher comorbidity burden, and more than half of the cohort was aged ≥ 70 years. Lymph node yield was higher in HLG (20 vs. 15; *p* < 0.001). Three‐year disease‐free survival (85.5% vs. 87.3%; *p* = 0.751) and overall survival (89.7% vs. 80.7%; *p* = 0.098) were comparable, with no differences in recurrence patterns.

**Conclusions:**

IMA ligation level does not significantly influence outcomes. However, in elderly or fragile patients, low ligation achieves very low leak rates without compromising oncological safety.

## 1 Introduction

Radical surgery represents the gold standard treatment for stages I‐III colorectal cancers. The primary tumor must be removed en bloc with the draining lymph nodes to achieve a curative intent. Vascular ligation is a fundamental surgical and oncological step of the procedure [1, 2].

When dealing with colorectal cancer, two levels of ligation are described and typically performed: “high ligation,” which consists of the ligation of the inferior mesenteric artery (IMA) at its root, and “low ligation,” distal to the bifurcation of the left colic artery, thus preserving it.

For patients with sigmoid cancer, high ligation of the IMA is considered the gold standard approach for two reasons: better oncological staging, due to a higher number of lymph nodes harvested, and lower risk of anastomotic tension, due to a greater mobility of the remaining colon [3]. However, high ligation may lead to reduced blood flow to the remaining colon, especially in old, frail patients, and to an increased risk of superior hypogastric plexus injuries during the dissection at the artery's root. Low ligation of the IMA, with or without lymph node dissection of the IMA root, on the other hand, is much more technically challenging, resulting in longer operative times [4]. Moreover, there are little data regarding the oncological outcomes [5].

Several studies have been proposed in the last decades to compare the two levels of vascular ligation, reporting inconclusive results in terms of oncological outcomes and post‐operative surgical results [6, 7]. Moreover, these studies are often heterogeneous regarding surgical technique, tumour stage, and frequently consider both sigmoid and rectal tumours together, despite the latter having different anatomy, biology, and oncological treatment approach [8, 9].

The aim of this study is to compare sigmoid resection with high or low ligation of the IMA in terms of oncological outcomes and post‐operative complications.

## 2 Materials and Methods

This is a retrospective multicentric study analysing patients who underwent surgical curative resection for sigmoid colon cancer at the Surgical departments of the E. Agnelli Hospital in Pinerolo, at the Colorectal Surgical Oncology Department of the National Institute of Cancer “IRCCS Pascale” in Naples and at the Department of General and Emergency Surgery of “A.O.U. Città della Salute e della Scienza” in Turin between January 2017 and December 2022. This study received approval from the local ethics committees. The study was conducted and reported in accordance with the STROBE (Strengthening the Reporting of Observational Studies in Epidemiology) checklist.

All consecutive patients (≥18 years old) with preoperatively staged I–III sigmoid cancer who underwent open or minimally invasive, elective or emergency surgical resection were included. Patients with distant metastases or preoperative evidence of tumor invasion of adjacent organs requiring multiorgan resection were excluded, as were patients who underwent protective stoma formation, Hartmann's procedure, or robot‐assisted surgery.

According to the surgical ligation level of the IMA, the patients were categorized into the high ligation (HLG) or low ligation group (LLG). In case of low ligation, lymphadenectomy of the IMA root was performed routinely. Postoperative management was the same for both groups, following the Italian ERAS guidelines [10]. This included early mobilization and oral intake, multimodal analgesia, avoidance of routine nasogastric tubes and drains, and standardized criteria for discharge.

Complete medical records of all patients considered in this study were collected using a prospectively maintained institutional database. The following variables were recorded for each patient:

– Preoperative data: sex, age, Body Mass Index (BMI), medical comorbidities (considered both individually and as a combined measure as none, one or more than two), American Society of Anaesthesiologists (ASA) score, tumor markers (CEA).
– Intraoperative data: surgical urgency, laparoscopic or open approach, and level of vascular ligation.
– Post‐operative data: 30‐day postoperative complications classified according to the Clavien‐Dindo system, based on the severity of the intervention required to manage each complication [11], presence of ileus, surgical site infection (according to the updated CDC guidelines [12]), or genitourinary complications, mortality, postoperative length of hospital stay (ICU and ward).
– Oncological data included histopathological characteristics and tumor staging, adjuvant therapy, and 3‐year outcomes: overall survival (OS), cancer‐specific survival (CSS), disease‐free survival, and recurrence rates (local and distant).
– Follow‐up data: readmission, reintervention.

The primary endpoint was the prevalence of local recurrence and distant metastases, disease‐free survival, and OS. Secondary outcomes included postoperative complications, including anastomotic leakage, and length of hospital stay.

### 2.1 Surgical Technique

All surgical procedures were performed by surgeons with extensive experience in colorectal surgery, defined as surgeons with a cumulative experience of more than 100 cases and an annual volume exceeding 40 procedures [13]. The initial phase of the procedure, whether performed open or with a laparoscopic approach, consisted of abdominal exploration to exclude hepatic metastases and peritoneal disease, and to confirm the presence of the sigmoid tumor without invasion of adjacent organs. Subsequent steps were identical in both the high‐ligation and low‐ligation groups: identification of the inferior mesenteric vein (IMV; level of ligation according to surgeon's preference), creation of the avascular plane between Toldt's and Gerota's fascia in a medial‐to‐lateral and cranio‐caudal direction, and identification of the IMA with lymphadenectomy at its origin. In the high‐ligation group, the IMA was ligated at its origin; in the low‐ligation group, the IMA was dissected from its origin, and the ligation was performed distal to the bifurcation of the left colic artery, thus preserving it. The procedure continued with mobilization of the descending‐sigmoid colon, with or without splenic flexure mobilization; transection of the upper rectum, specimen extraction, and perfusion assessment with indocyanine green (ICG) fluorescence imaging. In case of laparotomy, a lateral‐to‐medial approach was adopted, with colonic mobilization preceding vascular ligation. The anastomosis was performed using a mechanical stapler, followed by a bubble test. No drains were placed.

The choice between high‐ and low‐ligation was likely based on the individual surgeon's experience and intraoperative judgment; however, due to the retrospective design of the study, this variable was not systematically recorded, and available information was limited to operative reports. The three participating high‐volume centres followed a standardized surgical technique; ICG was routinely used in all cases (open and laparoscopic), no drains were placed, and no cases were excluded based on technical variations.

### 2.2 Statistical Analysis

Baseline, intraoperative, and postoperative characteristics were compared between high and LLGs. Quantitative variables, found non‐normally distributed by the Shapiro–Wilk test, were reported as median and interquartile range (IQR) and compared using the Mann–Whitney *U* test. Categorical variables were compared using the chi‐square or Fisher's exact test, as appropriate.

Univariable and multivariable logistic regression analyses were performed to assess the association between ligation level and dehiscence, adjusting for ASA score, age, and comorbidities. Univariable and multivariable logistic regression models were used to evaluate the association between ligation and cancer recurrence at 3 years, adjusting for postoperative chemotherapy and stage.

Kaplan–Meier survival analysis was used to estimate OS and CSS, and survival distributions were compared using the log‐rank test. Time zero for survival analysis was defined as the date of surgical intervention. Patients were followed from this point until death or censoring; no left‐censoring was present. Patients with missing data on the date of intervention, death, or last known follow‐up were excluded. OS was defined as the time from surgery to death from any cause or last follow‐up. CSS was defined as the time from surgery to death due to cancer, with deaths from other causes censored at the date of death. Within the 3‐year follow‐up period, deaths with incomplete date information were imputed conservatively: when only the year of death was available, the date was set to January 1 of that year (*n* = 2), and when only the month of death was available, the date was set to the first day of that month (*n* = 1).

Univariable and multivariable Cox proportional hazards models were used to assess the association between ligation and survival outcomes (both OS and CSS), adjusting for postoperative chemotherapy and stage.

The analyses were executed with STATA (v18) and SPSS (v29). Significance was set at *p*‐value < 0.050.

## 3 Results

### 3.1 Baseline Characteristics

Between January 2017 and December 2022, a total of 185 patients were included in the study: 127 (68.6%) were in the HLG and 58 (31.4%) in the LLG. Median age was 71 (IQR = 61−77.50), and 60% of the patients were males (Table 1). There were no statistically significant differences between groups in terms of age, sex, BMI, ASA classification, CEA values, and comorbidities.

**Table 1:** Baseline characteristics.

Variables	Sample (*n* = 185)	High ligation group (*n* = 127)	Low ligation group (*n* = 58)	*p*
Age (year)	71 (IQR 61−77.50)	71 (IQR 61−77)	71 (IQR 62.75−79)	*0.55*
Over 70 years old	101 (54.6%)	69 (54.3%)	32 (55.2%)	
*Sex*				*0.70*
Female	74 (40%)	52 (40.9%)	22 (37.9%)	
Male	111 (60%)	75 (59.1%)	36 (62.1%)	
BMI	25.71 (IQR 28.36–23.73)	25.60 (IQR 22.98−27.86)	26.87 (IQR 23.66−28.65)	*0.16*
*ASA*				*0.49*
1−2	98 (52.87%)	65 (52.4%)	33 (57.9%)	
3−4	83 (44.86%)	59 (47.6%)	24 (42.1%)	
*Number of comorbidities*				*0.83*
0	82 (45.3%)	58 (46.8%)	24 (42.1%)	
1	55 (30.4%)	37 (29.8%)	18 (31.6%)	
>2	44 (24.3%)	29 (23.4%)	15 (26.3%)	
*Comorbidities* a				
Diabetes	37 (20%)	27 (21.8%)	10 (17.5%)	*0.51*
Cardiac	59 (31.9%)	37 (29.8%)	22 (38.6%)	*0.24*
Pulmonary	19 (10.3%)	16 (12.9%)	3 (5.3%)	*0.12*
Urological	10 (5.4%)	6 (4.8%)	4 (7%)	*0.73*
Renal	6 (3.2%)	3 (2.4%)	3 (5.3%)	*0.38*
Metabolic	6 (3.2%)	3 (2.4%)	3 (5.3%)	*0.38*
Hepatic	5 (2.7%)	3 (2.4%)	2 (3.5%)	*0.65*
Vascular	5 (2.7%)	4 (3.2%)	1 (1.8%)	*1.00*
Collagenopaty	1 (0.5%)	0 (0%)	1 (1.8%)	*0.31*
CEA (ng/mL)	2.3 (IQR 1.6− 4)	2.3 (IQR 1.6−4.45)	2.2 (1.55−3.35)	*0.59*

*Note:* Italics indicate statistical significance (*p*‐values).

^a^ For each comorbidity only the ‘yes' category is shown; comorbidities are not mutually exclusive.

### 3.2 Intraoperative Characteristics

Most of the patients were approached laparoscopically: 77.2% in the HLG versus 79.3% in the LLG (*p* = 0.745), with comparable rates of conversion to open surgery (Table 2). Splenic flexure mobilization was significantly more frequent in the HLG (84.3%) than in the LLG (61.4%; *p* = 0.001). No significant differences between groups were observed in terms of operative time and estimated blood loss (Table 2).

**Table 2:** Intraoperative characteristics.

Variables	Sample (*n* = 185)	High ligation group (*n* = 127)	Low ligation group (*n* = 58)	*p*
Laparoscopic	144 (77.8%)	98 (77.2%)	46 (79.3%)	*0.74*
Converted to open	12 (6.5%)	7 (6%)	5 (9.4%)	*0.52*
Splenic flexure mobilisation	142 (76.7%)	107 (84.3%)	35 (61.4%)	***0.00***
Operation time (min)	143 (IQR 105−193.75)	135 (IQR 105−190)	150 (IQR 108−203.5)	*0.50*
Blood transfusion	10 (5.4%)	7 (5.5%)	3 (5.2%)	*1.00*

*Note:* Italics indicate statistical significance (*p*‐values), and bold to highlight the statistically significant results.

### 3.3 Postoperative Outcome and Pathology Findings

Length of hospital stay was comparable in both groups (HLG 6 days [IQR 7–4] vs. LLG 5 days [IQR 7.25–4]; *p* = 0.879). Overall, 93 patients (50.3%) developed postoperative complications, without significant difference between the HLG and LLG (49.6% vs. 51.7%, respectively; *p* = 0.669). Overall, most of the patients (41.1%) had grade 1−2 Clavien‐Dindo complications, without any significant difference between the two groups (*p* = 0.154). Overall, grade 3–4 complications occurred in 9.2% of the patients. Eight patients (4.9%) developed anastomotic leakage, 7 (5.5%) in the HLG versus 1 (1.7%) in the LLG (*p* = 0.438) (Table 3).

**Table 3:** Postoperative outcome and pathology findings.

Variables	Sample (*n* = 185)	High ligation group (*n* = 127)	Low ligation group (*n* = 58)	*p*
Hospital stay (days)	5 (IQR 4−7)	6 (IQR 4−7)	5 (4–7.25)	*0.88*
*Postoperative complications*				*0.67*
No	92 (49.7%)	64 (50.4%)	28 (48.3%)	
Yes	93 (50.3%)	63(49.6%)	30 (51.7%)	
*Dindo‐Clavien*				0.15
1–2	76 (41.1%)	49 (77.8%)	27 (90%)	
3−4	17 (9.2%)	14 (22.2%)	3 (10%)	
Dehiscence	8 (4.3%)	7 (5.5%)	1 (1.7%)	*0.44*
Reoperation	9 (4.9%)	8 (6.3%)	1 (1.7%)	*0.28*
Postoperative mortality (90 days)	2 (1.1%)	1 (0.8%)	1 (1.7%)	*0.53*
Readmission	2 (1.1%)	2 (1.6%)	0 (0%)	*1.00*
*T*				***0.01***
Tis‐T1	54 (29.2%)	28 (22%)	26 (44.8%)	
T2‐3	124 (67%)	93 (73‐2%)	31 (53.4%)	
T4	7 (3.8%)	6 (4.7%)	1 (1.7%)	
N				*0.09*
N1	36 (19.4%)	21 (16.5%)	15 (25.8%)	
N2	17 (9.2%)	15 (11.8%)	2 (3.4%)	
Stage				***0.04***
1	89 (48.1%)	55 (43.31%)	34 (58.6%)	
2	43 (23.2%)	36 (28.3%)	7 (12.1%)	
3	53 (28.6%)	36 (28.3%)	17 (29.3%)	
Lymph node harvested	19 (IQR 26−14)	20 (IQR 27−16)	15 (IQR 20.5−12)	***<0.00***
Specimen length (cm)	20 (IQR 23−15)	20 (IQR 25−15.7)	17 (IQR 20−15)	***<0.00***
Distance to margins (cm)	5 (IQR 7−3)	5 (IQR 7−3)	5 (IQR 6−3)	*0.14*
Adjuvant chemotherapy	35 (18.9%)	23 (18.1%)	12 (20.7%)	*0.75*

*Note:* Italics indicate statistical significance (*p*‐values), and bold to highlight the statistically significant results.

Among patients with stage 2−3 tumors, 24.3% underwent a high vascular ligation against 37.5% who underwent a low vascular ligation, however no statistically significant association was observed between the presence of vascular ligation and the number of comorbidities (*p* = 0.255).

T stage distribution in the overall sample showed a predominance of T2‐3 tumors (67%), with early‐stage tumors (Tis‐T1) more frequent in the LLG (44.8% vs. 22%, *p* = 0.006). Stage distribution differed significantly between groups (*p* = 0.039), with more Stage 1 cases in the LLG (58.6% vs. 43.3%) and more Stages 2−3 in the HLG. The number of lymph nodes harvested was significantly higher in the HLG than in the LLG (median 20 [IQR 27–16] vs. 15 [IQR 20.5–12]; *p* < 0.001).

In both univariable and multivariable logistic regression analyses, the level of vascular ligation was not significantly associated with anastomotic dehiscence (unadj. OR 0.30, 95% CI 0.04–2.50, *p* = 0.266; adj. OR 0.34, 95% CI 0.04–2.94, *p* = 0.328), even after adjusting for age, ASA score, and comorbidities. 90‐days postoperative mortality was low in both groups (HLG 0.8% vs. LLG 1.7%; *p* = 0.532), as was the rate of hospital readmission (HLG 1.6% vs. LLG 0%; *p* = 1.00) (Table 4).

**Table 4:** Anastomotic leakage.

	Univariable logistic regression		Multiple logistic regression	
	OR (95% CI)	*p*	adjOR (95% CI)	*p*
*Vascular ligation level*				
High	Ref.		Ref.	
Low	0.30 (0.04−2.50)	0.266	0.34 (0.04−2.94)	0.33
*Age ≥ 70*				
No	Ref.		Ref.	
Yes	2.59 (0.51−13.18)	0.252	4.77 (0.50−45.32)	0.17
ASA				
1−2	Ref.		Ref.	
3−4	1.19 (0.29−4.91)	0.810	0.81 (0.15−4.39)	0.81
*Comorbidities*				
No	Ref.		Ref.	
Yes	2.13 (0.40−11.27)	0.375	1.62 (0.002−0.12)	0.60

### 3.4 Long‐Term Outcomes

Follow‐up time was available for 115 patients in the HLG and for 57 patients in the LLG. Overall median follow‐up was 36 months (IQR = 33.98−36), 32.57 months in the HLG versus 30.66 months in the LLG (*p* = 0.221). Three‐year OS was slightly higher in the HLG (89.7% vs. 80.7%, *p* = 0.098), whereas cancer‐specific death was lower (5.1% vs. 10.7%, *p* = 0.206), but neither reached statistical significance. Local and distant recurrence rates were similar between the two groups of the study (Table 5).

**Table 5:** Oncological outcomes.

	High ligation group (*n* = 127)	Low ligation group (*n* = 58)	*p*
Time of FU (months)	53.9 (IQR 34.7−67.8)	47.6 (IQR 28.0−70.7)	*0.53*
3y‐overall survival	105 (89.7%)	46 (80.7%)	*0.10*
3y‐cancer specific death	6 (5.1%)	6 (10.7%)	*0.21*
3y‐disease‐free survival	94 (85.5%)	48 (87.3%)	*0.75*
3y‐recurrence rate	16 (14.5%)	7 (12.7%)	*0.75*
3y‐local recurrence	6 (5.4%)	3 (5.5%)	*1.00*
3y‐distant recurrence	10 (8.8%)	3 (5.3%)	*0.55*
Surgery of recurrence	9 (18.4%)	3 (8.1%)	*0.17*

*Note:* Italics indicate statistical significance (*p*‐values).

Considering recurrence occurrence, low ligation was not associated with recurrence in either univariable (OR 0.86, 95% CI 0.33–2.22, *p* = 0.751) or multivariable analysis (adjOR 1.04, 95% CI 0.37–2.89, *p* = 0.947) adjusted for adjuvant chemotherapy and stage. Postoperative chemotherapy showed a significant association in univariable analysis but not after adjustment, whereas stage 2 remained a significant predictor of recurrence in multivariable analysis (adj OR 4.19, 95% CI 1.09–16.02, *p* = 0.036).

**Figure 1:**
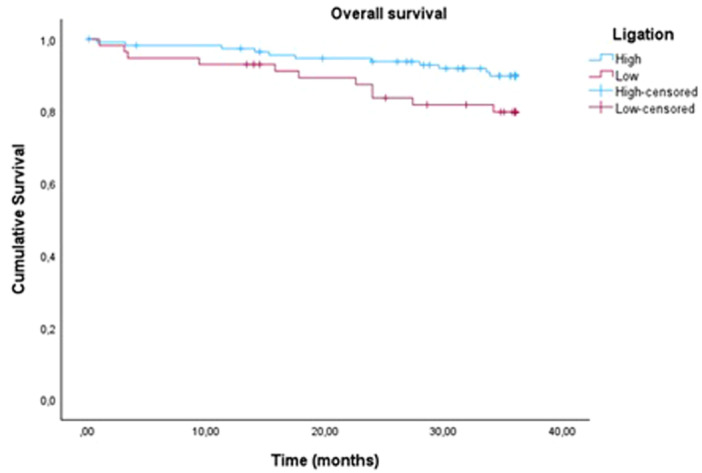
Kaplan–Meier curves showing OS according to the level of ligation (high vs low). The blue line represents high ligation, and the red line represents low ligation. Censored observations are indicated by crosses. OS is expressed as cumulative survival over time in months. OS, overall survival.

**Figure 2:**
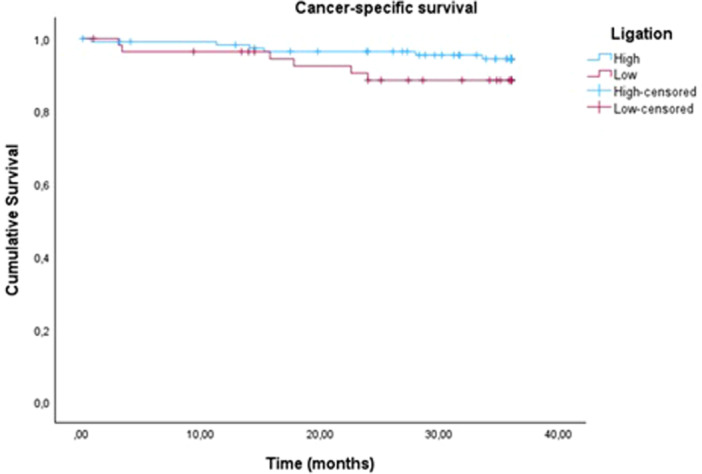
Kaplan–Meier curves illustrating CSS according to the level of ligation (high vs low). The blue line represents high ligation, and the red line represents low ligation. Censored observations are indicated by crosses. CSS is shown as cumulative survival over time in months. CSS, cancer‐specific survival.

During follow‐up, 11 deaths occurred in the HLG (9.6%) and 11 among those with LLG (19.3%). The survival distributions were not statistically significantly different (Figure 1 OS, log rank *χ* ^2^(1) = 3.329, *p* = 0.068). Among participants with follow‐up time, cancer‐specific death occurred in 6/115 patients (5.2%) in the HLG and in 6/56 patients (10.7%, one death was of unknown cause and therefore excluded) in the LLG (Figure 2 CSS, log‐rank *χ* ^2^(1) = 1.894, *p* = 0.169).

Cox regression analysis for OS showed that low vascular ligation was independently associated with a significantly higher risk of death (adjHR 2.49, 95% CI 1.03–5.98, *p* = 0.042) when adjusting for adjuvant chemotherapy and stage. In contrast, adjuvant chemotherapy and tumor stage were not significantly associated with mortality in either univariable or multivariable models (*p* > 0.05 for all comparisons).

Univariable and multivariable Cox regression analyses showed that low vascular ligation was not significantly associated with increased cancer‐specific death, with adjuvant chemotherapy (HR 2.26, 95% CI 0.38–13.54; *p* = 0.372), low ligation (HR 2.48, 95% CI 0.76–8.13; *p* = 0.133), and stage 2 and 3 disease all nonsignificant.

## 4 Discussion

Although high vascular ligation is traditionally considered the standard surgical approach in sigmoid colon cancer due to its potential oncological advantages, the preservation of the left colic artery through low vascular ligation has been proposed to maintain better perfusion of the remaining colon. This, in turn, could reduce the risk of postoperative complications such as anastomotic dehiscence. The optimal approach, however, remains a subject of ongoing debate. Kyung Ha Lee et al. [6] compared high‐ and low‐vascular ligation in a cohort of patients with sigmoid, rectosigmoid, and rectal tumors. They found no significant differences in distal margin clearance or the number of retrieved lymph nodes between the two groups, suggesting comparable oncological adequacy. However, the inclusion of rectal cancers may have introduced heterogeneity in the outcomes. More recent data from Wang et al. [7] involving 590 patients with sigmoid and rectal cancer provided stronger evidence favouring low vascular ligation. Low ligation was associated with a lower incidence of anastomotic leak and improved postoperative urinary function, without compromising long‐term oncological outcomes. A 2023 meta‐analysis by Reyaz et al. [9], encompassing 16 studies on colorectal cancer, further supported the oncological equivalence of the two techniques. Five‐year OS and disease‐free survival rates were comparable between high and low vascular ligation groups, although only one of the included studies focused exclusively on sigmoid cancer, limiting the applicability of these findings to this specific subgroup.

In our study, which focused exclusively on sigmoid colon cancer, anastomotic leak rates were low in both groups and even lower than the national averages reported by the Italian iCRAL [14] registry. Although not *statistically significant*, the lower anastomotic leak rate in the low vascular ligation group (1.7%) compared with the HLG (5.5%) is *clinically significant*, especially given that stage 2 and 3 patients in the LLG had a higher comorbidity burden (37.5% vs. 24.3%) and that more than half of the overall cohort was aged 70 years or older.

As expected, specimen length was significantly greater in the HLG. However, all patients in this study had resection margins exceeding 5 cm. In contrast to the findings reported by Kyung Ha Lee et al., a higher number of lymph nodes were retrieved in the HLG compared with the LLG (median 20 [IQR 27–16] vs. 15 [IQR 20.5–12]; *p* < 0.001). However, in the LLG lymphadenectomy was systematically carried out at the vessel origin, ensuring that the minimum criteria for adequate nodal harvest were met. In line with the findings of Ueno et al. [15], the regional pericolic nodes lie within 10 cm of the primary tumour and should be entirely included in the resection margin. Therefore, a modest reduction in the number of retrieved lymph nodes should not adversely affect oncological outcomes. These results are consistent with the data reported by the Italian Society of Surgical Oncology–Colorectal Cancer Network Collaborative Group [16], supporting from technical and oncological point of view less invasive and more tailored surgical approaches, such as segmental colonic resection, over extended but not superior procedures. Moreover, the multicenter randomized trial by Planellas et al. [17] comparing extended versus standard complete mesocolon excision in sigmoid colon cancer demonstrated that additional lymphadenectomy beyond the standard resection did not confer significant benefits in disease‐free or OS. In addition, in our cohort low vascular ligation was more commonly performed in patients with earlier‐stage disease, in whom limited lymphatic spread is expected.

Park et al. [8] reported shorter operative times in the LLG. The authors attributed this to the preservation of the left colic artery, which reduces the need for splenic flexure mobilization—a time‐consuming step in HLG procedures. In the present study no differences were found in terms of operation time even if there was a significantly lower rate of splenic flexure mobilization in the LLG. This result can be explained by the routinely performed lymph node dissection at the root of the IMA in the LLG in our cohort.

Unlike what was highlighted by Reyaz et al. [9], in the present study Cox regression analysis, even after adjusting for tumor stage and administration of adjuvant chemotherapy, showed a slightly better OS rates (*p* = 0.042) for patients undergoing high ligation. Several factors may contribute to this finding. First, the more frequent use of low vascular ligation in patients with multiple comorbidities may have introduced residual confounding, despite statistical adjustment. These patients may have had reduced physiological reserve or other unmeasured risk factors influencing long‐term outcomes. Second, the limited sample size may amplify the effect of these confounding variables, making the observed association less robust. Importantly, Cox‐regression analysis showed no significant differences in recurrence rates or CSS between the two groups, suggesting that the increased overall mortality observed with low vascular ligation might not be due to oncological failure, but potentially to non‐cancer‐related causes in a more comorbid population.

### 4.1 Limitations

The relatively small sample size may have limited the statistical power to detect significant differences in some outcomes, particularly for rare events such as anastomotic dehiscence. Furthermore, although data collection was performed using a prospectively maintained institutional database, the study design is retrospective, and the choice of high‐ versus low‐ligation was based on surgeon preference rather than a standardized protocol. This introduces the potential for selection bias, as certain surgeons or centers may routinely favour one technique over the other, independently of patient or tumor characteristics. Such preferences could be influenced by factors including tumor location, technical difficulty, or patient comorbidities, which may confound the comparison of outcomes between groups. Importantly, tumor location within the sigmoid colon (proximal, mid, or distal) was not always possible to determine with certainty, and stratification by segment could not be consistently performed. Additionally, differences in surgical expertise, perioperative care, or local institutional protocols may further affect results. These limitations should be carefully considered when interpreting the findings and their generalizability.

## 5 Conclusion

In response to the question of whether the level of vascular ligation in sigmoid surgery matters, our findings indicate that it does not. These results suggest that low vascular ligation can be a safe and oncologically sound alternative to high ligation in selected patients with sigmoid colon cancer. Particularly in early‐stage disease and in patients with significant comorbidities, low vascular ligation may offer reduced complication rates, especially regarding anastomotic leak rates, without compromising surgical or oncological efficacy. These conclusions should be interpreted in light of the study's retrospective design and potential selection bias.

## Funding

The authors have nothing to report.

## Ethics Statement

This research complies with the Wiley publishing ethics guidelines and was performed after obtaining approval from the Comitato Etico Territoriale (CET) Interaziendale A.O.U. Città della Salute e della Scienza di Torino (n.0001133, 18/03/2025).

## Conflicts of Interest

The authors declare no conflicts of interest.

## SYNOPSIS

In this multicenter retrospective study of 185 patients undergoing sigmoidectomy for colon cancer, high versus low inferior mesenteric artery ligation showed comparable short‐ and long‐term oncological outcomes. While high ligation yielded a greater lymph node harvest, low ligation preserved the left colic artery and achieved very low anastomotic leak rates, particularly in elderly or comorbid patients, without compromising disease control.

## Data Availability

The data that support the findings of this study are available on request from the corresponding author. The data are not publicly available due to privacy or ethical restrictions.
